# Silicious Deposit in the Urine, Supposed to Be from the Use of New Sponge

**Published:** 1853-03

**Authors:** Henry Johnson

**Affiliations:** Senior Physician to the Salop Infirmary


*Silicious Deposit in the Urine, supposed to be from the use of new sponge.* By Henry Johnson, M. D., Senior Physician to the Salop Infirmary. Silicic acid is a well-known constituent of natural urine and of urinary calculi; and it is always found in urinary concretions derived from brutes, according to Wurser and Lapaigne. But although, on the authority of these names, the occurrence of silex in urinary deposits and concretions may be deemed possible, this event is at any rate extremely rare, and, unless under peculiar circumstances, would be ascribed to accident, fraud, or mistake. I have, however, seen it, where the parties were far above all suspicion of any intention to deceive, and where the patient was too young and *girlish* to be the subject of that morbid *pruritus*, which probably leads to the *voluntary* introduction of foreign substances per vaginam.

As every case is of importance which proves a fact, or points out an obvious source of error, I think the following history is worth recording:— In 1850, I was desired to examine a small portion of white powder, which I understood was a deposit from urine; it had all the chemical characters of *silex*, and scratched glass with facility. I was informed, that notwithstanding every precaution having been taken to avoid error, more or less of this powder had been found at the bottom of the utensil for about a fortnight. I afterwards saw the patient. She was 11 years old, of spare habit, but tall for her age, and by no means strong. She was subject to sickness and anorexia; the bowels were torpid; the tongue was not clean; the catamenia had never appeared. At the time of my seeing her, the silicious deposit had ceased. The urine was pale, had a specific gravity, verging from 1011 to 1031, there was a light mucous cloud in it, and it contained some prismatic crystals of triple phosphate, and on another occasion urate of ammonia and a large quantity of urea.

The most distressing symptom was a most constant desire to pass water, by day and by night, so that she could not rest, and if the desire was not speedily gratified, the urine escaped involuntarily.

There appeared to be no organic disease present in this case, and the patient ultimately got well without any remarkable symptom or treatment. I feel quite satisfied that there was here no endeavor to impose upon her medical attendants, either on the part of the patient or her friends. The silicious deposit I have no doubt arose from the use of a *new sponge.* A silicious powder (quartz) is always found in sponge when new, and I have since seen a large quantity of this sort of dust, which had fallen out upon the shelf in a chemist's shop where these articles were kept; it is nearly pure silex, because sponges are prepared for sale by being soaked in dilute muriatic acid, which removes calcareous particles, and leaves such as are silicious.—*Prov. Med. and Sur. Jour.*

